# Identification and genetic evolutionary analysis of goose circovirus in Shandong and neighboring provinces in China in 2024

**DOI:** 10.1016/j.psj.2025.105826

**Published:** 2025-09-13

**Authors:** Xinao Yang, Yifan Wang, Peng Wu, Tao Zhu, Shuzheng Zhang, Xiaoyu Yang, Xiangfeng Meng, Ziping Jiang, Yuze Lu, Zhangyong Ning, Xiaowei Wu, Xingdong Song, Shijin Jiang, Liangmeng Wei

**Affiliations:** a Sino-German Cooperative Research Centre for Zoonosis of Animal Origin of Shandong Province, Shandong Provincial Key Laboratory of Zoonoses, College of Veterinary Medicine, Shandong Agricultural University, Tai'an, China; b College of Veterinary Medicine, South China Agricultural University, 483 Wushan Road, Guangzhou, 510642, China; c Guangzhou Customs Technology Center, Guangzhou, 510623, China

**Keywords:** Goose circovirus, Whole genome, Genetic evolution, Sequence-based analysis, Rep protein, Cap protein

## Abstract

Goose circovirus (GoCV), a significant pathogen impacting waterfowl health, has led to significant economic losses in the goose industry. Since it was first identified in Germany in 1999, it has spread globally. To investigate the prevalence, genetic characteristics, and variation patterns of GoCV in Shandong and its neighboring provinces in China, this study used PCR to screen GoCV-positive samples collected from diseased geese in Shandong, Hebei, Henan, and Anhui provinces. Ten positive samples were selected for whole-genome amplification and sequencing. The results revealed that the nucleotide sequence homologies of the entire genome, the Rep gene, and the Cap gene of the 10 GoCV strains varied in the ranges of 96.9–99.5%, 96.6–99.7%, and 96.8–99.3%, respectively; furthermore, the amino acid sequence homologies were observed to fall in the ranges of 94.4–99.3%, 99.3–100%, and 98.0–100%, respectively. The 10 GoCV isolates showed high homology with reference strains from Anhui, Shandong, Henan, and Zhejiang provinces, relatively low homology with reference strains from Heilongjiang and Guangdong, and low homology with three duck circovirus (DuCV) reference strains. The genetic evolution tree analysis showed that nine GoCVs were clustered within the GoCV-Ⅰa branch, while one was classified into the GoCV-Ⅰb branch. Notably, these branches were clearly distinct from those of the three DuCVs, indicating a distant phylogenetic relationship between GoCV and DuCV. The results of the amino acid sequence analysis showed that compared with the first GoCV isolate from the Chinese mainland, AY633653.1 yk1, the Rep and Cap proteins had mutations at the amino acid sites 4, 5, 47, 71 and 8, 26, 49, 59, 61, 74, 76, 78, 176, 186, respectively. The results of the three-dimensional structure prediction analysis showed that these proteins had high similarity in their overall configuration, but there were certain differences in some local regions. The data indicate that GoCV is present in parts of Shandong and its neighboring provinces. The Rep protein exhibits a high degree of conservatism, whereas the Cap protein demonstrates a high mutation rate. Local structural differences may influence viral virulence and immune escape capability; finally, GoCV is genetically distinct from DuCV. Continual monitoring and analysis of mutation trends in the virus are essential, as they play a critical role in the early prevention and control of goose circovirus disease.

## Highlights

• GoCV reference strains isolated from geographically adjacent regions exhibit high sequence homology, indicating the presence of extensive regional transmission pathways.
• Multiple GoCV subtypes coexist within the same geographic region, and certain strains demonstrate notable genetic divergence.
• The high homology between GoCV isolates from ducks and the experimental strains further confirms that recombination events and cross-species transmission of circoviruses occur frequently among avian species.

## Introduction

Goose circovirus (GoCV) is a member of the genus *Circovirus* in the family Circoviridae. It is non-enveloped and displays icosahedral symmetry. The diameter of GoCV virions ranges from 17–22 nm, making it one of the smallest viruses identified in geese thus far. GoCV is a covalently closed, single-stranded, negative-sense DNA virus with a genome size of approximately 2.0 kb. It encodes four major open reading frames (ORFs): ORF V1, ORF C1, ORF C2, and ORF V2. ORF V1 is located on the viral positive-sense strand (position 72/73nt-953nt) and encodes the Rep protein, which comprises 293 amino acids, exhibits high conservation in its amino acid sequence, displays good immunogenicity, and is essential for viral replication (Yao et al., 2022). ORF C1 is located on the negative-sense strand of the viral genome (position 1760/1761nt-1008nt) and encodes the Cap protein, which comprises 250 amino acids, forms the viral nucleocapsid, and serves as the major structural protein of the virus (Yao et al., 2022). The exact functions of ORF V2 and ORF C2 remain incompletely characterized.

GoCV exhibits similarities to other pathogens in the *Circovirus* genus, as several conserved motifs are present in the Rep protein, including FRLNN, HLQ, WWDGY, DDFYGWLP, and YCSK (Todd et al., 2001). Among these, three conserved motifs—namely FRLNN, HLQG, and YCSK—are hypothesized to play a role in viral roll-over replication (Todd et al., 2001). GoCV features a stem-loop structure located between ORF V1 and ORF C1, which contains a conserved nine-base sequence (TATTATTAC) on the stem-loop as well as two forward-repeating seven-base sequences (GTACTCC) and a pair of reverse-repeating sequences (GCTTCC) in close proximity to the stem-loop; these elements may potentially be involved in viral rolling-circle replication. One notable feature is the presence of a highly variable region downstream of the stem-loop structure, which is presumably associated with viral replication and virulence (Chen et al., 2003). The B-cell linear epitopes of the Cap protein in GoCV and duck circovirus (DuCV)—also in the *Circovirus* genus—are similarly positioned; however, the amino acid sequences of these two viruses differ significantly, leading to the hypothesis that there may be a difference in cross-protective efficacy between GoCV and DuCV (Chen et al., 2020).

GoCV was first discovered and reported by Soike in Germany in 1999 (Snoike et al., 1999). Since then, scholars in Hungary, Taiwan (China), Chongqing municipality, Jiangxi province, Guangdong province, Shandong province, and Poland have detected GoCV. In 2005, Yu Xuping et al. detected GoCV in geese that died from avian influenza in Yongkang, Zhejiang Province, in the first report of GoCV on the Chinese mainland (Yu et al., 2005). The genetic evolution tree indicates that GoCV can be divided into three populations: GoCV-I, GoCV-II, and GoCV-III. Notably, GoCV-I comprises the two subpopulations GoCV-Ia and GoCV-Ib, and GoCV-II consists of the two subpopulations GoCV-IIa and GoCV-IIb. The three GoCV genome lengths are 1820 bp, 1821 bp, and 1822 bp. As of the completion date (May 25, 2025), there were 32 GoCVs with 1820-bp genomes, 114 GoCVs with 1821-bp genomes, and 26 GoCVs with 1822-bp genomes, for a total of 172 strains deposited in the Gene Bank.

Clinical signs of GoCV infection in geese typically include stunted growth, disheveled plumage, weight loss, lethargy, and depression. In severe cases, feather loss and necrosis of the feather follicle may be observed (Ting et al., 2021). It has been demonstrated that GoCV can cause systemic infections in geese and that high levels of viral DNA may be present in sick geese (Smyth et al., 2005). Histopathologically, GoCV is frequently detected in the spleen, thymus, bone marrow, liver, and kidneys of geese; the main manifestations include pathological changes such as reduction in lymphocytes and histiocytes in tissues like the spleen and thymus, foci of lymphocytic inflammation in the liver, interstitial pneumonitis with lymphocytic infiltration in the lungs, nephritis, and granulomatous degeneration of renal cells (Guo et al., 2011). GoCV primarily infects the goose immune system, resulting in immune system damage, reduced immunity, and secondary infections; accordingly, clinical cases of single GoCV infections in geese are relatively rare (Todd, 2000). GoCV cannot be cultured using conventional methods, such as cell culture, chicken embryo inoculation, or experimental animal models, which limits further investigation into GoCV.

In this study, we amplified the whole genome of positive samples detected in geese suspected of being infected with GoCV from Shandong and its neighboring provinces in China. We conducted homology comparisons and constructed genome-wide phylogenetic trees based on the nucleotide sequences of the whole genome. Additionally, we constructed phylogenetic trees for the deduced amino acid sequences of the Rep and Cap proteins, performed amino acid homology comparisons, and analyzed amino acid mutation sites. These findings will provide a valuable reference for the effective prevention and control of GoCV in Shandong and its neighboring provinces.

## Materials and methods

### Sources of disease material

In 2024, laboratory pathogen testing was conducted on diseased goose samples collected from Shandong, Anhui, Hebei, and Henan provinces. Positive samples identified as GoCV were stored at -80°C in a freezer for further analysis.

### GoCV primer design and synthesis

Primers were synthesized based on the GoCV identification; full-length amplification primer sequences are presented in Table 1. In this study, the GoCV-F/R primers used for identification were synthesized by referring to the primer sequences published by Shuqi Xu (Xu et al., 2024a). All primers were synthesized by Qingdao Qikexin Biotechnology Co., Ltd., a biotechnology company in China.

**Table 1:** Primer sequences used in this study.

Name of primer	Sequences of primers	Length of amplified fragment (bp)
GoCV-F	AGGGATTCCTGAGTCTGCGA	451
GoCV-R	ACAACCACATCCTGCCCATTAT	
GoCV-F1	CGTCTGTATCGTCGTCTCCG	830
GoCV-R1	CATCACAACCACATCCTGCC	
GoCV-F2	CTCCAGTGATCTCTCCGACG	1406
GoCV-R2	GCCTGTAACGGTTTCTGTCC	

### Preliminary characterization of the pathogen

Liver, spleen, and lung tissue samples collected for disease material analysis were mixed, cut into small pieces, transferred to sterilized centrifuge tubes, and processed by adding steel beads and a sodium chloride solution. The samples were then ground in a grinder at 60 Hz for 6 min and subsequently centrifuged at 10,000 rpm for 2 min. The supernatant was collected, and DNA was extracted using a DNA extraction kit, then amplified using GoCV-F/R primers in the following 20-µL reaction system: 10 µL of 2 × Rapid Taq Master Mix, 2 µL of DNA template, 1 µL each of forward and reverse primers, and 6 µL of ddH₂O. The amplification procedure went as follows: initial denaturation at 95°C for 3 min, 35 cycles of denaturation at 95°C for 15 s each, annealing at 55°C for 15 s each, and extension at 72°C for 2 min, followed by a final extension at 72°C for 5 min. The PCR products were analyzed through 1% agarose gel electrophoresis.

### GoCV whole genome detection and sequencing

The whole genome was amplified using the primers GoCV-F1/R1 and GoCV-F2/R2, and using pathogen-positive clinical samples confirmed by diagnostic primers as the DNA template. The amplification system consisted of 10µL of 2 × Rapid Taq Master Mix, 2 µL of DNA template, 1 µL each of forward and reverse primers, and 6 µL of ddH_2_O, for a total volume of 20µL. The amplification procedure consisted of the following steps: initial denaturation at 95°C for 3 min, 35 cycles of denaturation at 95°C for 15 s each, annealing at 55°C for 15 s each, and extension at 72°C for 2 min, followed by a final extension at 72°C for 5 min. The PCR products were analyzed through 1% agarose gel electrophoresis, and the target bands were recovered using a DNA gel recovery kit. The purified PCR products were then sent to Sangon Bioengineering (Shanghai) Co., Ltd. for sequencing.

### Analysis of GoCV whole gene sequence and genetic evolution

The sequencing results obtained through the above-described processes were processed using DNAStar software, to assemble the full-length sequence of the gene. After performing sequence comparison with NCBI (National Center for Biotechnology Information), the correctly sequenced GoCV gene sequences were aligned with known sequences from GenBank. Phylogenetic tree construction and similarity analysis were conducted using MEGA11 software. Additionally, DNAStar software was used to analyze the whole genome of GoCV, as well as its Rep and Cap genes, for nucleotide and amino acid sequence similarity to reference strains. BioEdit software was used to analyze the amino acid mutation sites in the structural Cap protein and the functional Rep protein. General information and accession numbers of the reference strains obtained from GenBank are provided in Supplementary Table 1.

## Results and analyses

### Results of GoCV PCR assay identification

Through the PCR-based identification of diseased goose samples, the results indicate that amplification products of the specific primers appeared at 451 bp in 10 of the tested samples; these products were confirmed by sequencing and were identified as GoCV. The 10 viral strains were named GoCV/1-655/SD/2024(PV877095), GoCV/3-667/SD/2024(PV877096), GoCV/4-671/SD/2024(PV877097), GoCV/5-674/SD/2024(PV877098), GoCV/6-675/SD/20249(PV877099), GoCV/7-678/SD/2024(PV899100), GoCV/8-679/SD/2024(PV899101), GoCV/9-681/AH/2024(PV899102), GoCV/10-683/HB/2024(PV899103), and GoCV/11-685/HN/2024(PV899104). Detailed information on the 10 isolates is shown in Table 2.

**Table 2:** 10 GoCV strains information.

Name of Isolate	Source
GoCV/1-655/SD/2024	Dezhou, Shandong, China
GoCV/3-667/SD/2024	Liaocheng, Shandong, China
GoCV/4-671/SD/2024	Heze, Shandong, China
GoCV/5-674/SD/2024	Liaocheng, Shandong, China
GoCV/6-675/SD/2024	Liaocheng, Shandong, China
GoCV/7-678/SD/2024	Dezhou, Shandong, China
GoCV/8-679/SD/2024	Dezhou, Shandong, China
GoCV/9-681/AH/2024	Suzhou, Anhui, China
GoCV/10-683/HB/2024	Xingtai, Hebei, China
GoCV/11-685/HN/2024	Xinyang, Henan, China

### Results of GoCV whole genome assay and sequencing

The amplification of the collected samples using the two pairs of primers mentioned in GoCV-F1/R1 and GoCV-F2/R2 yielded target fragments of 830 bp and 1406 bp, respectively, which were consistent with the expected band sizes. It was determined that the 10 strains belonged to two genotypes, GoCV-Ⅰ and GoCV-Ⅱ, and the whole genome length of all 10 GoCV isolates was 1821 nt.

### Results of genome-wide homology comparison and genetic evolution analysis of GoCV

**Fig. 1:**
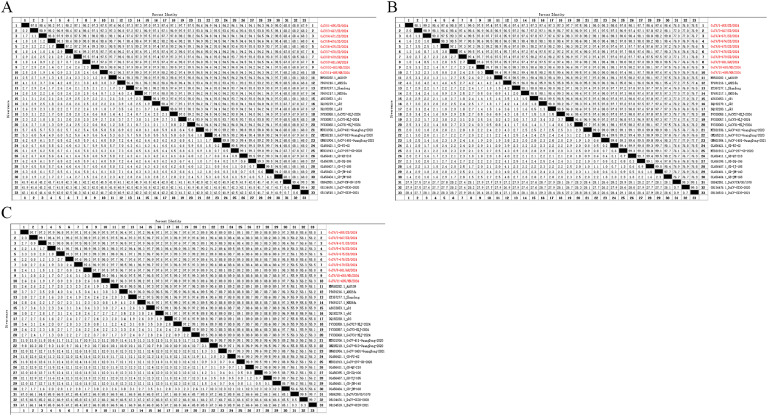
Comparison of nucleotide sequence homology between 10 goose circovirus strains and reference strains. Fig. 1A shows the results of whole-genome nucleotide homology analysis.Fig. 1B shows the results of Rep protein nucleotide homology analysis. Fig. 1C shows the Results of Cap protein nucleotide homology analysis.The nucleotide sequences of 10 GoCV strains were processed using DNASTAR software to obtain the complete genome, Rep gene, and Cap gene sequences. Corresponding whole genome, Rep, and Cap sequences of reference strains were retrieved from the NCBI database and similarly processed using DNASTAR software. All nucleotide sequence homology analyses were performed using MegAlign, employing the ClustalW Multiple Sequence Alignment program for sequence alignment.

**Fig. 2:**
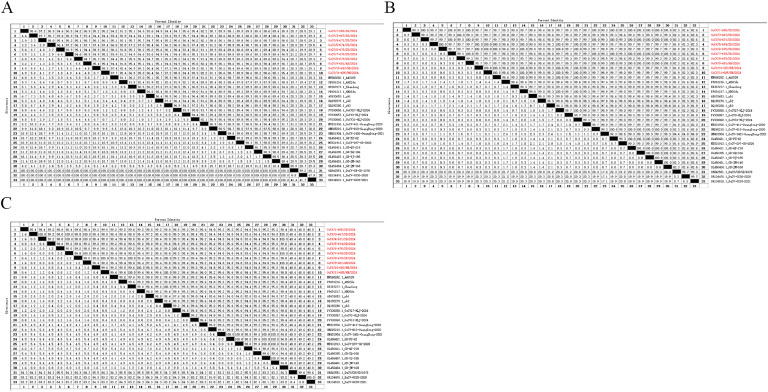
Comparison of amino acid sequence homology between 10 goose circovirus strains and reference strains. Fig. 2A shows the results of whole-genome amino acid homology analysis. Fig. 2B shows the results of Rep protein amino acid homology analysis. Fig. 2C shows the results of Cap protein amino acid homology analysis.The nucleotide sequences of the complete genome, Rep gene, and Cap gene from the 10 GoCV strains and reference strains were translated into amino acid sequences using DNASTAR software. The resulting amino acid sequences for Rep and Cap were aligned using the ClustalW Multiple Sequence Alignment program within MegAlign software. The complete genome amino acid sequences were aligned using the Jotun Hein method to generate homology results.

As shown in Fig. 1A, the genome-wide nucleotide similarity of the 10 GoCV strains ranged from 96.9% to 99.5%. Among these, GoCV/10-683/HB/2024 and GoCV/11-685/HN/2024 exhibited the closest genetic relationship, with the highest homology at 99.5%. The homologies between the 10 GoCV strains and the HN20du Henan strain are high, ranging from 97.1% to 99.6%; in contrast, the homologies with the GoCV-297-GD-2020 Guangdong strain are the lowest, ranging from 93.4% to 94.4%. The nucleotide homologies between the 10 viral strains and the reference strains from the Anhui, Shandong, Henan, Zhejiang, Heilongjiang, and Guangdong provinces of China fell in the ranges of 95.9–99.2%, 97.2–98.4%, 97.1–99.6%, 96.9–98.2%, 96.9–99.5%, and 93.4–98.8%, respectively. In addition, the homologies with the three DuCV reference strains ranged from 67.7% to 68.5%. All 10 GoCV strains encoded 606 amino acids. As shown in Fig. 2A, the results of the amino acid homology comparison indicated that the genome-wide amino acid homology ranged from 94.4% to 99.3%, with the highest homology (99.3%) observed between GoCV/10-683/HB/2024 and GoCV/11-685/HN/2024. The amino acid homologies between the 10 viral strains and the reference strains from Anhui, Shandong, Henan, Zhejiang, Heilongjiang, and Guangdong provinces fell in the ranges of 91.3–97.7%, 94.4–97.2%, 94.1–99.3%, 93.1–97.2%, 93.4–99.3%, and 88.3–98.0%, respectively. Additionally, the amino acid homologies with the three DuCV reference strains ranged from 19.9% to 29.7%.

**Fig. 3:**
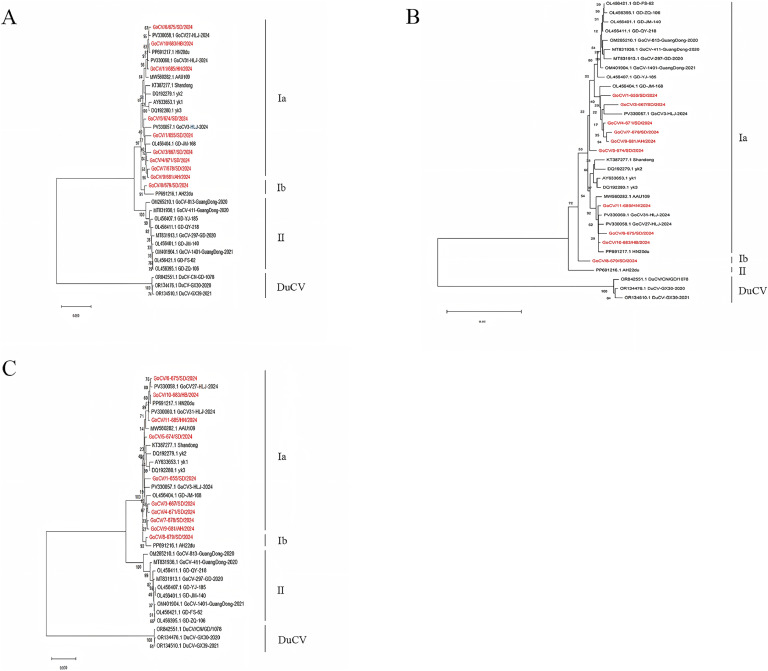
Phylogenetic tree of GoCVs: whole gene (A), Rep gene (B), Cap gene (C). All the sequences involved in the phylogenetic trees were aligned using ClustalW using the neighbor-joining method (1000 replicates) in MEGA11.

The results of the genome-wide genetic evolution tree for the 10 GoCV strains are shown in Fig. 3A. All GoCV strains except for GoCV/8-679/SD/2024 clustered within the GoCV-Ia branch, together with the AAU109 strain from Anhui province and the GD-JM-168 strains from Shandong, Henan, Zhejiang, Heilongjiang, and Guangdong provinces. In contrast, GoCV/8-679/SD/2024 clustered within the GoCV-Ib branch, together with the AH22du strain. GoCV/3-667/SD/2024, GoCV/4-671/SD/2024, GoCV/7-678/SD/2024, and GoCV/9-681/AH/2024 are located on the same branch and exhibit close genetic relationships; similarly, GoCV/6-675/SD/2024, GoCV/10-683/HB/2024, and GoCV/11-685/HN/2024 cluster together on another branch with close genetic relationships, and GoCV/1-655/SD/2024 and GoCV/5-674/SD/2024 cluster together on yet another branch. The 10 GoCV strains and the remaining reference strains are located on different branches from the three DuCV reference strains and exhibit distant genetic relationships.

### Results of rep protein homology comparison and genetic evolution analysis for GoCV

The full length of the Rep gene in the 10 GoCV isolates was 882 nucleotides. As shown in Fig. 1B, the nucleotide homologies of the Rep protein among these isolates ranged from 96.6% to 99.7%; among them, GoCV/10-683/HB/2024 and GoCV/11-685/HN/2024 exhibited the highest homology at 99.7%, indicating a high degree of similarity and indicating that they may belong to the same or similar genetic groups. The Rep nucleotide homologies of the 10 viral strains with the reference strains from Anhui, Shandong, Henan, Zhejiang, Heilongjiang, and Guangdong provinces fell in the ranges of 95.6–99.4%, 97.2–98.3%, 96.7–99.5%, 96.9–98.6%, 96.3–99.5%, and 96.9–99.0%, respectively. In contrast, the homologies with the three DuCV reference strains ranged from 76.2% to 77.1%.

The Rep proteins from all 10 GoCV isolates consist of 293 amino acids.As shown in Fig. 2B, the results of the amino acid homology comparison indicated that the Rep proteins of the 10 GoCV isolates exhibited amino acid homologies ranging from 99.3% to 100%. This finding indicates that the strains are also highly similar at the protein level, further supporting the results of the nucleotide homology analyses. The amino acid homologies between the isolates and the reference strains were high, yet slightly lower than among the isolates themselves: for example, GoCV/3-667/SD/2024 exhibited 99.3% homology with both GoCV/4-671/SD/2024 and GoCV/9-681/AH/2024, indicating only minor differences between these strains.

The results of the genetic evolution tree for the 10 GoCV Rep genes are shown in Fig. 3B. Except for GoCV/8-679/SD/2024, all GoCV strains clustered within the GoCV-Ia branch together with the reference strains from Anhui (AAU109 virulent strain), Shandong, Henan, Zhejiang, Heilongjiang, and Guangdong provinces. In contrast, GoCV/8-679/SD/2024 belonged to the GoCV-Ib branch. GoCV/4-671/SD/2024 is located on the same branch as and exhibits close genetic relationships with GoCV/7-678/SD/2024 and GoCV/9-681/AH/2024. GoCV/3-667/SD/2024 is located on the same branch as and exhibits a close genetic relationship to GoCV3-HLJ-2024. GoCV/1-655/SD/2024 clusters on the same branch as the five aforementioned strains, with which it exhibits a close genetic relationship, whereas GoCV/5-674/SD/2024 is also clustered within the GoCV-Ⅰa branch.. GoCV/6-675/SD/2024, GoCV/10-683/HB/2024, and GoCV/11-685/HN/2024 are located on the same branch and exhibit close genetic relationships. In addition, the 10 GoCV strains and the remaining reference strains were not clustered on the same branch as the three DuCV strains and exhibited distant genetic relationships.

### Homology comparison of GoCV cap protein and genetic evolution analysis results

The full length of the Cap protein gene among the 10 GoCV isolates was 753 nucleotides. As shown in Fig. 1C, the results of the nucleotide homology comparison revealed that the nucleotide homologies of the Cap protein among the 10 GoCV isolates ranged from 96.8% to 99.3%; the highest homology (99.3%) was observed between GoCV/6-675/SD/2024 and GoCV/10-683/HB/2024, demonstrating high similarity in their Cap gene sequences. The nucleotide homologies of the Cap gene between the 10 GoCV strains and the reference strains from Anhui, Shandong, Henan, Zhejiang, Heilongjiang, and Guangdong provinces fell in the ranges of 96.0–98.7%, 97.1–98.4%, 96.9–99.7%, 96.4–98.4%, 96.7–99.3%, and 88.2–98.8%, respectively. The nine isolates other than GoCV/3-667/SD/2024 exhibited relatively high homologies with the Anhui, Shandong, Henan, Zhejiang, Heilongjiang, and Guangdong isolates, whereas GoCV/3-667/SD/2024 showed higher homology with the Guangdong isolate (GD-JM-168). Notably, all 10 GoCV strains demonstrated the lowest homology and highest divergence with the three DuCV strains.

The Cap gene of all 10 GoCV isolates encoded 250 amino acids. As shown in Fig. 2C, the results of the amino acid homology comparison indicated that the amino acid homology of the Cap proteins among the 10 GoCV isolates ranged from 98.0% to 100%. This finding demonstrates that these isolates also exhibited a high degree of similarity at the protein level, further supporting the results of the nucleotide homology analysis. Among these, 100% homology was observed between GoCV/3-667/SD/2024 and GoCV/4-671/SD/2024, GoCV/7-678/SD/2024, and GoCV/9-681/AH/2024; between GoCV/4-671/SD/2024 and GoCV/7-678/SD/2024 as well as GoCV/9-681/AH/2024; between GoCV/7-678/SD/2024 and GoCV/9-681/AH/2024; and between GoCV/8-679/SD/2024 and GoCV/11-685/HN/2024, indicating complete sequence similarity. The 10 GoCV isolates exhibited low homology with the three DuCV strains, ranging from 48.0% to 48.8%.

The results of the genetic evolution tree for the 10 GoCV Cap gene strains are presented in Fig. 3C. All 10 GoCV Cap gene strains clustered within the same GoCV-Ia branch as the reference strains from Anhui, Shandong, Henan, Zhejiang, and Heilongjiang provinces. GoCV/6-675/SD/2024 and the Heilongjiang isolate GoCV27-HLJ-2024 are co-clustered within the same sub-branch, exhibiting a relatively close genetic affinity. GoCV/10-683/HB/2024 is located on the same sub-branch as the Henan isolate HN22du, to which it exhibits close genetic relatedness. GoCV/11-685/HN/2024 is clustered on the same branch as the aforementioned four strains and shows close genetic relatedness. GoCV/5-674/SD/2024 is grouped on the same branch as the Shandong strains (Shandong) and the Zhejiang strains (yk1, yk2, yk3), demonstrating close genetic relatedness. Both GoCV/3-667/SD/2024 and GoCV/4-671/SD/2024, along with GoCV/7-678/SD/2024 and GoCV/9-681/AH/2024, are clustered within the same phylogenetic branch, which are all closely related. GoCV/8-679/SD/2024 is clustered on the same branch as and shows close genetic relatedness to the Anhui strain (AH22du). In addition, the 10 GoCV strains and the remaining reference strains were not clustered on the same branch as the three DuCV strains and exhibited distant genetic relatedness.

### Amino acid mutation analysis and protein structure prediction of Rep and Cap proteins

The results of the amino acid mutations in the rep proteins of the 10 GoCV strains, shown in Table 3, revealed four mutation sites—at positions 4, 5, 47, and 71—compared with the first GoCV isolate from mainland China (i.e., AY633653.1 yk1). At position 4, strains GoCV/1-655/SD/2024, GoCV/4-671/SD/2024, GoCV/7-678/SD/2024, and GoCV/9-681/AH/2024 had the N→S mutation; at position 5, all GoCV strains except GoCV/3-667/SD/2024 and GoCV/8-679/SD/2024had the G→S mutation; at position 47, all 10 GoCV strains had the D→E mutation; and at position 71, all 10 GoCV strains had the V→E mutation.

**Table 3:** Comparison of Amino Acid Mutations in the Rep Protein Relative to the first isolated strain of GoCV (AY633653.1 yk1) in mainland China.

Designation of strains	Amino acid locus			
	4	5	47	71
GoCV/1-655/SD/2024	S	S	E	E
GoCV/3-667/SD/2024	N	G	E	E
GoCV/4-671/SD/2024	S	S	E	E
GoCV/5-674/SD/2024	N	S	E	E
GoCV/6-675/SD/2024	N	S	E	E
GoCV/7-678/SD/2024	S	S	E	E
GoCV/8-679/SD/2024	N	G	E	E
GoCV/9-681/AH/2024	S	S	E	E
GoCV/10-683/HB/2024	N	S	E	E
GoCV/11-685/HN/2024	N	S	E	E
AY633653.1 yk1	N	G	D	V

The results of Cap protein amino acid mutation (see Table 4) showed that, compared with the first GoCV isolate in mainland China (AY633653.1 yk1), subsets of the 10 GoCV isolates had mutations at amino acid position 8, 61, 74, 76, 176, and 186—specifically, at amino acid position 8, the P→Q mutation; at position 61, the D→E mutation; at position 74, the P→S mutation; at position 76, the S→T mutation; at position 176, the I→V mutation; and at position 186, the T→S mutation. Additionally, all 10 GoCV isolates had mutations at amino acid position 26 (K→R), 49 (S→N), 59 (G→S), and 78 (A→T).

**Table 4:** Comparison of Amino Acid Mutations in the Cap Protein Relative to the first isolated strain of GoCV (AY633653.1 yk1) in Mainland China.

Designation of strains	Amino acid locus									
	8	26	49	59	61	74	76	78	176	186
GoCV/1-655/SD/2024	Q	R	N	S	D	P	S	P	I	T
GoCV/3-667/SD/2024	P	R	N	S	E	S	T	P	I	T
GoCV/4-671/SD/2024	P	R	N	S	E	S	T	P	I	T
GoCV/5-674/SD/2024	P	R	N	S	E	P	S	P	I	T
GoCV/6-675/SD/2024	P	R	N	S	D	P	S	P	V	S
GoCV/7-678/SD/2024	P	R	N	S	E	S	T	P	I	T
GoCV/8-679/SD/2024	P	R	N	S	D	P	S	P	I	T
GoCV/9-681/AH/2024	P	R	N	S	E	S	T	P	I	T
GoCV/10-683/HB/2024	P	R	N	S	D	P	S	P	V	T
GoCV/11-685/HN/2024	P	R	N	S	D	P	S	P	I	T
AY633653.1 yk1	P	K	S	G	D	P	S	A	I	T

**Fig. 4:**
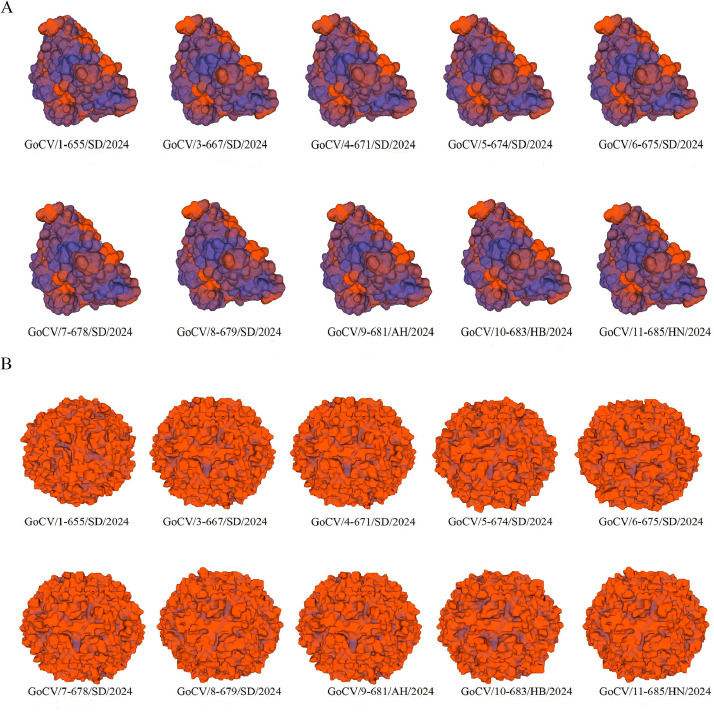
Protein structure prediction. Fig. 4A shows the Rep protein structure prediction. Fig. 4B shows the Cap protein structure prediction.

In this study, the three-dimensional structures of the Rep and Cap proteins from the 10 GoCV isolates were predicted using bioinformatics methods. The results, depicted in Fig. 4, revealed that these protein structures exhibit high similarity in overall conformation, implying that they likely possess similar functions and biological properties and demonstrate high genetic conservation. Nevertheless, slight variations were observed among the strains in certain local regions of both Rep and Cap proteins, indicating the presence of some genetic diversity. In comparison with the first GoCV isolate from mainland China (AY633653.1 yk1), all 10 GoCV isolates had mutations in specific amino acid sites of the Rep protein, which may affect their DNA-binding ability, enzyme activity, or protein-protein interactions. Additionally, according to the amino acid homology analyses, the Cap protein structures of the 10 GoCV viruses exhibited a high degree of similarity, indicating that these strains are structurally closely genetically related; however, local structural differences may potentially influence the infectivity, stability, or host range of the viruses.

## Discussion

Among the 10 strains from Shandong and some neighboring provinces in this study, the whole-genome and Rep gene nucleotide and amino acid homologies were slightly higher than those between the isolates and the reference strains from Anhui, Shandong, Henan, and Zhejiang. However, certain individual strains exhibited greater homology to the reference strains in their Cap gene sequences; for instance, GoCV/10-683/HB/2024 showed higher homology to the regional reference strains than GoCV/5-674/SD/2024 and GoCV/7-678/SD/2024. Nevertheless, the 10 isolates exhibited slightly lower homologies with the strains from the Guangdong region. All these findings indicate the presence of the presence of certain genetic differences among these strains.

Comparative analysis of the whole genomes of the 10 GoCV strains and the first GoCV isolate in mainland China, AY633653.1 yk1, showed that the nucleotide homologies ranged from 96.9 to 97.9% and the amino acid homologies ranged from 93.1 to 96.4%. Compared with the three reference strains of DuCV, the nucleotide homologies were 67.7–68.5% and the amino acid homologies were 19.9–29.7%, and they were not in the same branch. These results indicate that since the discovery of the first strain of GoCV in mainland China, a general genetic mutation of GoCV has occurred and other branches of GoCV have been introduced into mainland China; in addition, they show that the genetic differences of the circoviruses between ducks and geese are farther away from each other, with obvious species differentiation. Among the 10 GoCV strains, the genome-wide nucleotide homologies of GoCV from Shandong ranged from 96.9% to 99.0%, while the genome-wide amino acid homologies ranged from 93.6% to 98.2%; these results suggest the presence of different isoforms of GoCV within Shandong. Notably, the homologies of GoCV/3-667/SD/2024 and GoCV/6-675/SD/2024 were the lowest, indicating the existence of GoCVs with significant genotypic differences within the same geographical region. Additionally, GoCV/10-683/HB/2024 and GoCV/11-685/HN/2024 exhibited the highest homology, indicating a close genetic relationship between GoCV strains from Hebei and Henan, which may be attributed to frequent poultry trade and breed introduction between the two provinces. Furthermore, the 10 GoCV strains showed generally higher homologies with reference strains from Hebei, Henan, Anhui, and Shandong, suggesting potential extensive regional transmission pathways for GoCV within these areas.

Previous studies have demonstrated that regional factors serve as one of the key bases for GoCV classification and that recombination acts as a major driver of GoCV diversification (Yao et al., 2022; Yu et al., 2007). Stenzel et al. (Stenzel et al., 2018) identified the presence of recombinant strains and recombination events of GoCV in migratory birds, indicating GoCV transmission and recombination complexity. Previous studies have also documented the genetic heterogeneity of circoviruses prevalent in waterfowl (Liao et al., 2022). Recently, Shuqi Xu et al. (Xu et al., 2024a) reported for the first time the detection of GoCV in duck flocks in China, which they hypothesized might result from recombination of GoCV facilitated by migratory birds during trans-regional migrations. The same research team has recently identified DuCV in goose flocks (Xu et al., 2024b), providing additional evidence of the widespread recombination and cross-host transmission of circoviruses among bird species. This was supported by the observation in this study that the reference strains AH22du and HN20du, both isolated from ducks in Anhui and Henan provinces, exhibited high nucleotide homologies (95.9–98.0% and 97.1–99.6%, respectively) and amino acid homologies (91.3–95.1% and 94.1–99.3%, respectively) with the 10 GoCV strains included in the data analysis.

ORF V1 encodes the Rep protein, which plays a critical role in GoCV replication. The Rep protein contains several conserved motifs—including FRLNN, HLQG, and YCSK— that are associated with rolling-circle replication. ORF C1 encodes the Cap protein, which exhibits strong immunogenicity. Extant studies have demonstrated that the N-terminal region of the Cap protein contains a nuclear localization signal (NLS) sequence and a continuous arginine sequence, and the Cap protein also harbors five B-cell linear epitopes: α (28 aa-47 aa), β (129 aa-148 aa), γ (105 aa-124 aa), δ (156 aa-175 aa), and ε (231 aa-250 aa) (Chen et al., 2024). These findings imply that the similarity of the B-cell linear epitopes among the Cap proteins of different avian circoviruses could be leveraged for the development of GoCV vaccines based on cross-immunity. In a comparison of the sequences of the Rep and Cap genes, the nucleotide and amino acid homologies of the Rep protein among the 10 GoCV strains analyzed in this study were found to be higher than those of the Cap protein. This suggests that the Rep protein is more evolutionarily conserved than the Cap protein, with the ORF C1 gene exhibiting higher genetic diversity than the ORF V1 gene. Accordingly, the highly conserved sequence region of the Rep gene can serve as a target in establishing PCR-based diagnostic methods for GoCV (Yang et al., 2020). Compared with the Rep protein, the Cap protein exhibits more amino acid mutation sites, indicating that the Cap gene is prone to high-frequency mutations. This is likely related to the distinct functions of the Rep and Cap proteins; since the proteins encoded by the Cap gene primarily serve as nucleocapsid proteins and can induce specific immune responses in the host organism, it is hypothesized that Cap proteins may evade host immune surveillance and defense mechanisms through high-frequency mutations, or potentially co-evolve with the host (Xu et al., 2024).

The initial symptoms of GoCV infection are not readily apparent, which is consistent with the seasonal pattern of higher incidences of goose diseases in winter and spring and lower incidences in summer and autumn (Niu et al., 2018). However, GoCV infection in geese leads to the suppression of host immune function and facilitates immune escape, thereby increasing susceptibility to secondary infections. GoCV targets the host's bursa of Fabricius, leading to a marked decrease in the number of lymphocytes within the organ—one of the contributing factors to the reduction in host immunity (Guo et al., 2011). Goose infections with GoCV are often accompanied by co-infections with one or more pathogens, such as goose parvovirus, goose reovirus, goose Tembusu virus (Niu et al., 2018), and *Escherichia coli*. Therefore, elucidating the immunosuppressive and immune escape mechanisms of GoCV is crucial for developing effective prevention and control strategies as well as for vaccine design.

GoCV is challenging to prevent and control, as its isolation and culture are particularly difficult: to date, there has been no official report of successful isolation and culture of GoCV strains worldwide. This has resulted in limited research on the pathogenic mechanisms of GoCV, and so current prevention and control strategies primarily focus on preventive measures. An isolate of GoCV was obtained by Chen Jialong et al. (Chen et al., 2024) in Guangdong; however, it could not be used to develop a traditional vaccine (inactivated or attenuated vaccine) because the virus was only capable of maintaining replication at a low number of passages and with low titers.

In recent years, research on GoCV vaccines has made some progress. For instance, Chen Jialong et al. from Guangdong expressed the Cap protein exogenously and developed a subunit vaccine with adjuvant (Chen et al., 2024); additionally, some studies have demonstrated that a DNA vaccine can be constructed using the GoCV Cap gene (Blanchard et al., 2003). However, due to genetic variation in GoCV—particularly the high-frequency mutations in the Cap gene—the cross-protection rate of the vaccine remains low and requires further improvement. In future studies, multivalent vaccines targeting different genotypes of GoCV can be developed based on the aforementioned characteristics, to achieve the desired protection rate. At the same time, a study conducted by Jidang Chen in Guangdong (Chen et al., 2021) indicates that GoCV may achieve immune escape through the loss of the recognition site for B-cell neutralizing antibodies; consequently, the B-cell epitope could serve as a critical target for further research and development of vaccines or other specific immunoprophylactic products. In terms of transmission, a study by Shao Zhen and Diao Youxiang (Shao and Diao, 2023) revealed that GoCV viral nucleic acids were readily detectable in geese across all seasons and age groups on goose farms; notably, higher levels of GoCV were detected in chicks, indicating the possibility of vertical transmission. Therefore, it is essential to strengthen the purification of GoCV in breeding flocks as well as enhance measures for introductions, quarantine, and the control of transmission pathways and vectors.

The epidemiological pattern of GoCV is evolving with the global development of large-scale and intensive goose farming. Thus, to provide support for further research on GoCV genes, in this study we analyzed the genetic evolutionary characteristics of GoCV strains in some regions of Shandong and its neighboring provinces. Nevertheless, the pathogenicity differences among various genotypes, the mechanisms of virus–host immune system interactions, and the optimization of vaccines and therapeutic strategies remain areas that require further exploration. Therefore, continual monitoring and research into its transmission dynamics and mutation trends are essential.

## Funding

This work was supported by the 10.13039/501100001809National Natural Science Foundation of China (32473019) and the Key Research and Development Program of Shandong Province, China (2022CXPT005).

## CRediT authorship contribution statement

**Xinao Yang:** Writing – original draft, Software, Data curation, Formal analysis. **Yifan Wang:** Writing – original draft, Software, Methodology. **Peng Wu:** Software, Methodology, Formal analysis. **Tao Zhu:** Software, Resources. **Shuzheng Zhang:** Software, Investigation. **Xiaoyu Yang:** Validation, Supervision. **Xiangfeng Meng:** Formal analysis, Data curation. **Ziping Jiang:** Methodology, Investigation. **Yuze Lu:** Validation, Supervision. **Zhangyong Ning:** Visualization, Validation. **Xiaowei Wu:** Investigation. **Xingdong Song:** Writing – review & editing, Validation. **Shijin Jiang:** Writing – review & editing, Project administration. **Liangmeng Wei:** Writing – review & editing, Resources, Project administration, Conceptualization.

## Disclosures

The authors declare that they have no conflicts of interest.

## Appendix Supplementary materials

Image, application 1

## Footnotes

Supplementary material associated with this article can be found, in the online version, at doi:10.1016/j.psj.2025.105826.
