# Supplementary material for: Identification and genetic evolutionary analysis of goose circovirus in Shandong and neighboring provinces in China in 2024
Source: Poult Sci. 2025 Sep 13;104(11):105826. doi: 10.1016/j.psj.2025.105826 (PMC12466204; doi:10.1016/j.psj.2025.105826)
Supplement: Supplementary file 1 [file mmc1.docx]

**Supplementary Table 1.** Reference strains information

| Strains | GenBank  Accession No. | Years | Host | **Registered Address** |
| --- | --- | --- | --- | --- |
| AAU109 | MW560282.1 | 2020 | Goose | Hefei,Anhui,China |
| AH22du | PP691216.1 | 2022 | Duck | Nanyang,Henan,  China |
| Shandong | KT387277.1 | 2015 | Goose | Tai'an,Shandong,  China |
| HN20du | PP691217.1 | 2020 | Duck | Nanyang,Henan,  China |
| yk1 | AY633653.1 | 2007 | Goose | Hangzhou,Zhejiang,China |
| yk2 | DQ192279.1 | 2007 | Goose | Hangzhou,Zhejiang,China |
| yk3 | DQ192280.1 | 2007 | Goose | Hangzhou,Zhejiang,China |
| GoCV27-HLJ-2024 | PV330058.1 | 2024 | Goose | Harbin,Heilongjiang,China |
| GoCV3-HLJ-2024 | PV330057.1 | 2024 | Goose | Harbin,Heilongjiang,China |
| GoCV31-HLJ-2024 | PV330060.1 | 2024 | Goose | Harbin,Heilongjiang,China |
| GoCV-411-GuangDong  -2020 | MT831936.1 | 2020 | Goose | Foshan,Guangdong,China |
| GoCV-813-GuangDong  -2020 | OM265210.1 | 2020 | Goose | Foshan,Guangdong,China |
| GoCV-1401-GuangDong  -2021 | OM401904.1 | 2021 | Goose | Foshan,Guangdong,China |
| GD-FS-62 | OL456421.1 | 2021 | Goose | Foshan,Guangdong,China |
| GoCV-297-GD-2020 | MT831913.1 | 2020 | Goose | Foshan,Guangdong,China |
| GD-QY-218 | OL456411.1 | 2021 | Goose | Foshan,Guangdong,China |
| GD-ZQ-106 | OL456395.1 | 2021 | Goose | Foshan,Guangdong,China |
| GD-YJ-185 | OL456407.1 | 2021 | Goose | Foshan,Guangdong,China |
| GD-JM-140 | OL456401.1 | 2021 | Goose | Foshan,Guangdong,China |
| GD-JM-168 | OL456404.1 | 2021 | Goose | Foshan,Guangdong,China |
| DuCV-CN-GD-1078 | OR842551.1 | 2023 | Duck | Foshan,Guangdong,China |
| DuCV-GX30-2020 | OR134476.1 | 2020 | Duck | Nanning,Guangxi,  China |
| DuCV-GX39-2021 | OR134510.1 | 2021 | Duck | Nanning,Guangxi,  China |
